# A Screen for Round Egg Mutants in *Drosophila* Identifies Tricornered, Furry, and Misshapen as Regulators of Egg Chamber Elongation

**DOI:** 10.1534/g3.111.001677

**Published:** 2012-03-01

**Authors:** Sally Horne-Badovinac, Joseph Hill, Gary Gerlach, William Menegas, David Bilder

**Affiliations:** * Department of Molecular Genetics and Cell Biology, University of Chicago, Chicago, Illinois 60637; † Department of Molecular and Cell Biology, University of California, Berkeley, California 94720

## Abstract

The elongation of tissues and organs during embryonic development results from the coordinate polarization of cell behaviors with respect to the elongation axis. Within the *Drosophila melanogaster* ovary, initially spherical egg chambers lengthen dramatically as they develop to create the elliptical shape of the mature egg. This morphogenesis depends on an unusual form of planar polarity within the egg chamber’s outer epithelial cell layer known as the follicle cells. Disruption of follicle cell planar polarity leads to the production of round rather than elongated eggs; however, the molecular mechanisms that control this tissue organization are poorly understood. Starting from a broadly based forward genetic screen, we have isolated 12 new round egg complementation groups, and have identified four of the mutated genes. In mapping the largest complementation group to the *fat2* locus, we unexpectedly discovered a high incidence of cryptic *fat2* mutations in the backgrounds of publicly available stocks. Three other complementation groups correspond to the genes encoding the cytoplasmic signaling proteins Tricornered (Trc), Furry (Fry), and Misshapen (Msn). Trc and Fry are known members of an NDR kinase signaling pathway, and as a Ste20-like kinase, Msn may function upstream of Trc. We show that all three proteins are required for follicle cell planar polarity at early stages of egg chamber elongation and that Trc shows a planar polarized distribution at the basal follicle cell surface. These results indicate that this new mutant collection is likely to provide novel insight into the molecular mechanisms controlling follicle cell planar polarity and egg chamber elongation.

The lengthening of tissues and organs is a common theme in the development of multicellular organisms. This type of directional morphogenesis often requires that cell behaviors be coordinately polarized within the tissue plane and with respect to the elongation axis. The Frizzled/Strabismus and Fat/Dachsous planar cell polarity (PCP) pathways align cell behaviors during many tissue elongation events. However, recent work in *Drosophila* has shown that directional morphogenesis can also be controlled by alternate planar polarity systems (Bertet *et al.* 2004; Gutzeit *et al.* 1991; Taniguchi *et al.* 2011; Zallen and Wieschaus 2004). One of these alternate systems is the unusual mode of planar polarity that transforms the initially spherical egg chamber into a highly elongated egg.

Egg chambers are multicellular units within the ovaries of adult female flies that will each give rise to a single egg. They are composed of 15 nurse cells and one oocyte, surrounded by an epithelium of about 850 follicle cells. Though initially spherical, egg chambers lengthen dramatically along their anterior-posterior (A-P) axes as they develop. This elongation coincides with a planar polarization of the follicle cell epithelium that is best seen by examining dense, parallel arrays of actin bundles at the basal surface of each cell. These bundles align with one another and perpendicular to the A-P axis, a pattern that is mirrored by extracellular matrix (ECM) molecules in the adjacent basement membrane (Gutzeit 1990; Gutzeit *et al.* 1991). The result is a striking circumferential organization of structural molecules around the outside of the egg chamber, orthogonal to the axis of elongation. The prevailing model is that these molecular assemblies then act as a “molecular corselet” that limits isometric egg chamber growth to promote A-P elongation. Further light has been shed on this process by the recent discoveries that egg chamber rotation and oscillating contractions of the basal follicle cell surfaces contribute to elongation morphogenesis (Haigo and Bilder 2011; He *et al.* 2010). However, the molecular mechanisms that establish and maintain follicle cell planar polarity have remained elusive.

The little that we do know about the molecular mechanisms controlling follicle cell planar polarity and egg chamber elongation has largely come from work on mutations that produce round rather than elongated eggs. These studies have shown that cell-ECM interactions play a central role in this system, by identifying ECM components, such as Collagen IV, Laminin, and Perlecan, as well as ECM receptors, like Integrins, Dystroglycan, and Lar (Bateman *et al.* 2001; Deng *et al.* 2003; Frydman and Spradling 2001; Gutzeit *et al.* 1991; Haigo and Bilder 2011; Mirouse *et al.* 2009; Schneider *et al.* 2006). Cell-cell communication may also be required, as mutations in the atypical cadherin Fat2 produce a particularly strong round egg phenotype (Viktorinova *et al.* 2009). Many of these early round egg mutations were identified via reverse genetic or candidate gene approaches. However, the frequency with which this phenotype has appeared by chance suggests that forward screening could be used to genetically dissect this process.

Starting from a broadly based screen for mutations that disrupt epithelial polarity and morphogenesis in the follicle cells, we have isolated 12 new round egg complementation groups and identified four of the mutated genes. We first describe the mapping of the largest complementation group to the *fat2* gene, as these experiments revealed a surprisingly high incidence of cryptic *fat2* mutations in the backgrounds of publicly available stocks. We then show that three other complementation groups correspond to the cytoplasmic signaling proteins Tricornered (Trc), Furry (Fry), and Misshapen (Msn). Trc and Fry are members of a known NDR kinase signaling pathway and, as a Ste20-like kinase, Msn may function upstream of Trc. All three proteins are required for follicle cell planar polarity during early stages of egg chamber elongation, and Trc shows a planar polarized distribution at the basal follicle cell surface. These results indicate that this new mutant collection is likely to provide significant molecular insight into the mechanisms controlling follicle cell planar polarity and egg chamber elongation.

## MATERIALS AND METHODS

### *Drosophila* stocks

Follicle cell clones were generated as previously described (Duffy *et al.* 1998) using the *e22c-Gal4* driver and enhanced GFP under the control of the ubiquitin promoter (*ubi-eGFP*) to mark wild-type cells. Mutant alleles used for complementation and phenocopy analysis include *LanA^9-32^ FRT79* (Deng and Ruohola-Baker 2000), *rhea^1^ FRT80*, *ilk^1^*, *fbl^1^*, *fbl^DG04702^*, *fry^1^ FRT80*, *trc^1^ FRT80*, *msn^102^ FRT80*, and *msn^172^ FRT80*. All are available from the Bloomington *Drosophila* Stock Center unless otherwise indicated.

### EMS mutagenesis

Male flies carrying an isogenized FRT80 chromosome were starved for eight hours and subsequently fed a 25 mM EMS solution overnight at room temperature. The mutagenized males were then mated *en masse* to *w*; *TM3/TM6b* females. Single F1 males of the genotype *w*; *FRT80/TM3 were each crossed to four females of the genotype *w*; *e22c-Gal4*, *UAS-Flp/CyO*; *ubi-eGFP*, *FRT80*. Approximately five F2 females of the genotype *w*; *e22c-Gal4*, *UAS-Flp/ +*; *FRT80/ubi-eGFP, *FRT80* were collected from each cross for ovary dissection and staining (see below). Mutant chromosomes were recovered by crossing F2 sibling males to *w*; *TM3/TM6b* females.

### Immunohistochemistry and microscopy

All females were fed yeast for three days to stimulate egg production. Ovaries were dissected in PBS and fixed at room temperature in 4% EM-grade formaldehyde (Polysciences) in PBS. For the screen, egg chambers were stained with Phalloidin-TRITC (1:500, Sigma) and DAPI (1:1000) in PBS with 0.1% triton. Guinea pig anti-Trc [1:600, Emoto *et al.* (2004)] and rabbit anti-Fry [1:400, He *et al.* (2005b)] antibody stains were performed in PBS with 0.3% triton, followed by incubation with AlexaFluor-488–labeled secondary antibodies (Invitrogen). All egg chambers were mounted in SlowFade antifade solution (Invitrogen). Images were obtained using either a Leica TCS SL or a Zeiss LSM 510 confocal microscope, and processed with Adobe Illustrator and Photoshop.

## RESULTS

### A screen for mutations that disrupt epithelial polarity in the follicle cells

**Figure 1:**
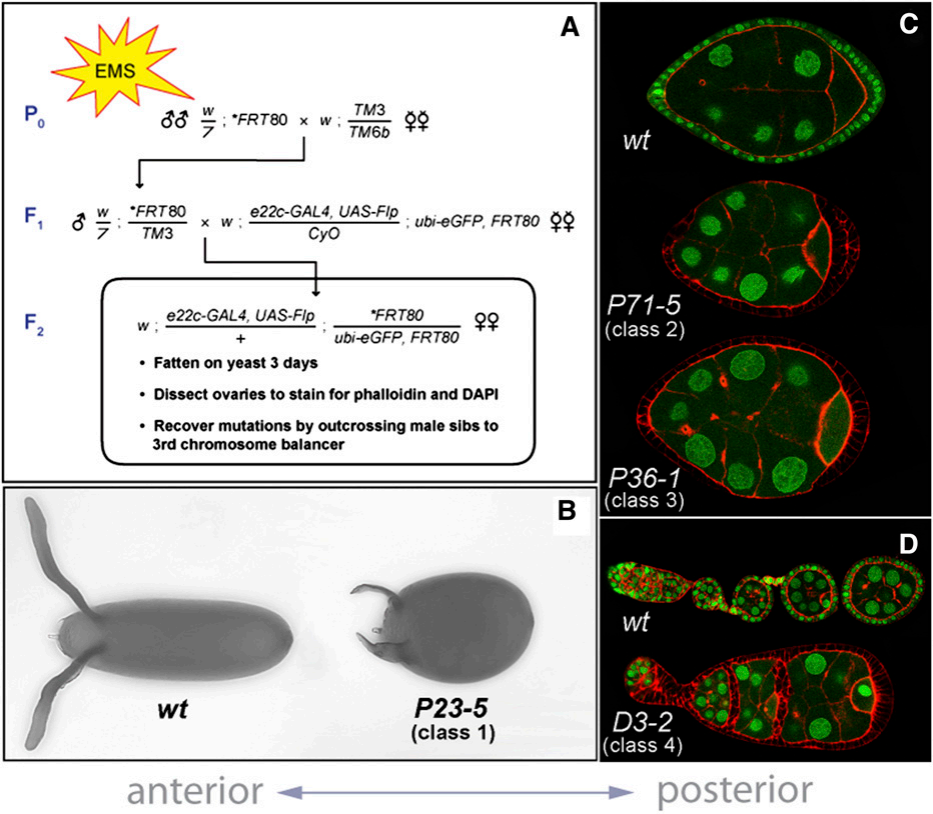
Overview of the mosaic follicle cell screen for epithelial defects. (A) Basic screening strategy. (B–D) Examples of the four major phenotypic classes. (B) Class 1: Round eggs. (C) Classes 2 and 3: Loss of apical-basal polarity (*P71-5*) and epithelial gaps (*P36-1*). (D) Class 4: Germ cell encapsulation defects. (C–D) All cells are outlined with phalloidin (red), and wild-type cells are marked with GFP (green). In all panels, anterior is to the left.

To identify new genes required for epithelial polarity and morphogenesis in the follicle cells, we used the directed mosaic technique to screen random mutations on the left arm of the third chromosome (3L) (Duffy *et al.* 1998). We used EMS to mutagenize male flies carrying an isogenized FRT80 chromosome, and then mated these males *en masse* to virgin females carrying a third chromosome balancer. Individual, balanced F1 males, each carrying independent mutations on the FRT80 chromosome, were then crossed to virgin females of a tester line that expresses the Flp recombinase under the control of the *e22c-Gal4* driver and contains an FRT80 chromosome marked with ubiquitously expressed GFP (Figure 1A). Mosaic ovaries were dissected from F2 females, and then individual egg chambers were visually inspected, using the lack of GFP to identify mutant clones and Phalloidin and DAPI to reveal epithelial morphology. Using this strategy, we screened 4929 mutagenized chromosomes and identified 61 individual mutations affecting multiple aspects of follicle cell form and function (Figure 1 and Table S1).

The mutations we identified fall into four main phenotypic classes. In order of prevalence, these include: (1) production of round eggs, (2) loss of apical-basal polarity/monolayer organization, (3) epithelial gaps, and (4) defects in germ cell encapsulation. An example of each of these four phenotypic classes is shown in Figure 1. There are also six individual mutations that disrupted other aspects of follicle cell biology (Table S1). Within each category, we performed pair-wise crosses between the individual mutations to establish complementation groups (Table 1 and Table S1). Of the 47 groups identified, 32 contain a single allele, suggesting that the screen was not saturating. We have previously reported our work on three of the apical-basal polarity mutants: *avalanche*, *Dhc64c*, and *Glued* (Horne-Badovinac and Bilder 2008; Lu and Bilder 2005). We have now turned our attention to the “round egg” class, as this new mutant collection will likely provide significant insight into the poorly understood form of planar polarity operating within the follicle cells.

**Table 1:** The round egg complementation groups

Complementation Group	Alleles
RE-A (*fat2*)	6
RE-B (*fry*)	4
RE-C	2
RE-D (*trc*)	1
RE-E (*msn*)	1
RE-F	1
RE-G	1
RE-H	1
RE-I	1
RE-J	1
RE-K	1
RE-L	1

The round egg phenotype was particularly striking during the screen because the defective eggs were visible during ovary dissection and did not require subsequent staining and mounting of egg chambers onto slides. Moreover, greater than one third of the mutations identified (21/61) fell into this class. Through pair-wise crosses, we found that these mutations form 12 complementation groups, which we have named “RE-” followed by the letters A–L (Table 1). Given the known requirement for cell-ECM interactions in controlling egg shape (Bateman *et al.* 2001; Frydman and Spradling 2001; Gutzeit *et al.* 1991), we performed complementation tests between representative alleles from each of the 12 RE groups and three candidate genes on chromosome 3L: *Laminin A*, *rhea*/*talin*, and *Integrin-linked kinase*. In all cases, our RE mutations complemented these alleles (data not shown). These results suggested that we had identified 12 new loci required for fly egg shape. Although we are just beginning the molecular characterization of these mutants, we report our initial findings for 4 of the RE loci below.

### The mapping of RE-A revealed cryptic *fat2* mutations in publicly available stocks

**Figure 2:**
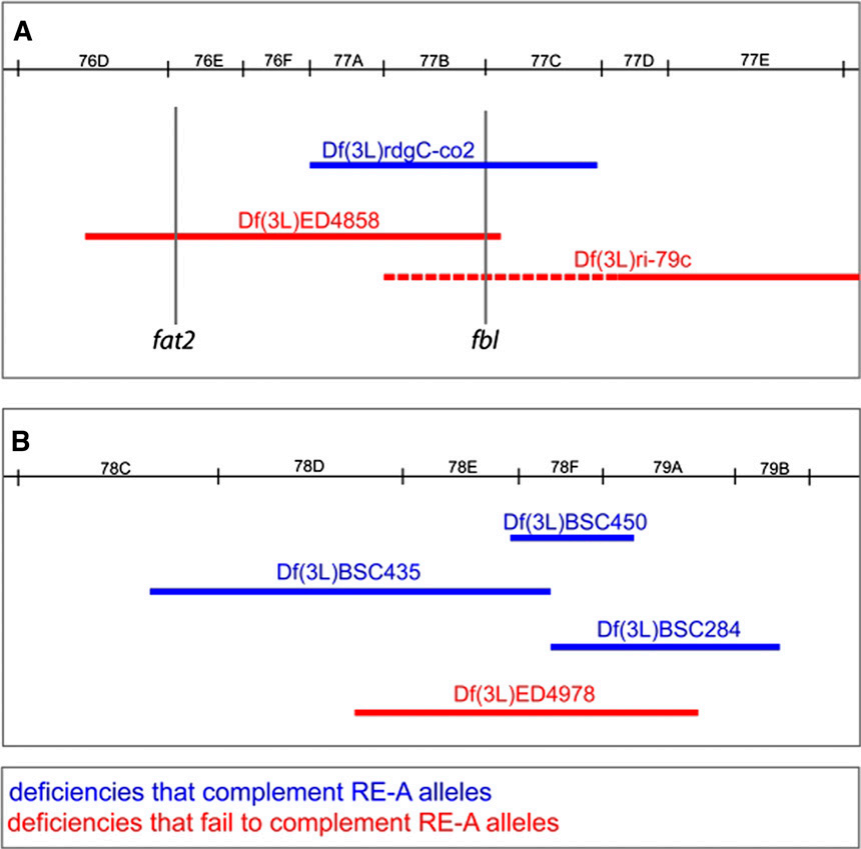
Deficiency mapping of the RE-A complementation group. (A) The first chromosomal region identified by deficiency mapping, containing the *fat2* and *fbl* genes. (B) The second chromosomal region identified by deficiency mapping. For each region, select deficiencies are shown that either complement (blue) or fail to complement (red) the RE-A alleles.

To identify the gene disrupted by the RE-A complementation group, we employed a deficiency mapping strategy. In *trans*-heterozygous combinations, these six alleles produce viable females that are sterile due to the production of round eggs. Consequently, we crossed the *J18* allele to a panel of 3L deficiency stocks, and looked for round eggs in hemizygous females. This initial screen identified two nonoverlapping deficiencies, *Df(3L)ED4858* (76D3-77D1) and *Df(3L)ED4978* (78D5-79A2), that failed to complement *J18* (Figure 2, A and B). Screening three additional deficiencies that completely tiled *Df(3L)ED4978* revealed that none produced round eggs with *J18* (Figure 2B). However, when we performed the complementary experiment with the other chromosomal region, a single overlapping deficiency, *Df(3L)ri-79C*, produced round eggs with *J18* (Figure 2A). We then performed complementation tests with individual mutant alleles that map to the 77B-77C1 overlapping region. This experiment revealed that *fumble^1^* (*fbl^1^*), a lesion in the pantothenate kinase gene, failed to complement *J18*. We subsequently showed that all six RE-A alleles produced round eggs in combination with the two overlapping deficiencies and *fbl^1^*.

Although these data were consistent with the RE-A round egg phenotype being due to mutations in *fbl*, several observations argued against this hypothesis. First, neither a second *fbl* allele (*fbl^DG04702^*) nor a third overlapping deficiency [*Df(3L)rdgC-co2*] produced round eggs in combination with *J18*. Second, sequencing the *fbl* coding region in the RE-A allele *N103-2* revealed no lesions. Third, while this work was in progress, Viktorinova *et al.* (2009) reported that females mutant for the atypical cadherin gene *fat2*, which is located within the region uncovered by the *Df(3L)ED4858* deficiency (Figure 2A), produced round eggs.

To ask whether the RE-A complementation group corresponded to *fat2*, we sequenced this gene in all of the mutant backgrounds and found premature stop codons within the *fat2* coding region of five of the six RE-A alleles (Table 2). Surprisingly, we also found premature stop codons in the *fat2* coding regions in the *Df(3L)ri-79c* and *fbl^1^* backgrounds (Table 2). We did not find a coding region lesion in *Df(3L)ED4978*, but this deficiency fails to complement all of the *fat2* alleles, suggesting that this background harbors a *fat2* mutation as well. Together these data indicate that the RE-A complementation group corresponds to *fat2*. They also revealed a surprisingly high incidence of cryptic *fat2* alleles in the backgrounds of publicly available stocks.

**Table 2:** New *fat2* alleles

Allele	Lesion
*fat2^C19-2^*	Unknown
*fat2^G58-2^*	Q762 to stop
*fat2^J18^*	S2841 to stop
*fat2^N103-2^*	Complex lesion leading to a premature stop codon at position 3718
*fat2^O13-3^*	Q3782 to stop
*fat2^Q21-5^*	L1845 to stop
*fat2^fbl1^*	Complex lesion leading to a premature stop codon at position 691
*fat2^Df(3L)ri-79c^*	E720 to stop
*fat2^Df(3L)ED4978^*	Unknown

### Tricornered, Furry, and Misshapen are regulators of egg chamber elongation

Although deficiency mapping proved problematic for RE-A (*fat2*), this method easily identified the gene corresponding to our second largest complementation group, RE-B. The deficiency mapping itself localized the RE-B locus to 67B10-67C5, and subsequent complementation tests with known mutant alleles in this region revealed that the four RE-B alleles all failed to complement *furry^1^* (*fry^1^*). We then confirmed that loss of *fry* caused the round egg phenotype by identifying premature stop codons in the *fry* coding regions of three of the four RE-B alleles (Table 3) and by showing that *fry^1^* follicle cell clones produce round eggs (data not shown). Recently, Fang *et al.* (2010) showed that a *UAS-fry* transgene expressed under *Actin-GAL4* control can rescue *fry^1^*/*fry^2^* null mutant flies to adulthood but that the resulting females are sterile due to the production of shorter eggs. These data indicate that Fry is the egg shape regulator disrupted by the RE-B complementation group.

**Table 3:** New *trc*, *fry*, and *msn* alleles

Allele	Lesion
*trc^K23^*	W395 to stop
*fry^H33-2^*	Unknown
*fry^O31^*	Q1008 to stop
*fry^O41^*	W394 to stop
*fry^O98-4^*	G666 to stop
*msn^P23-5^*	Q1379 to stop

Fry is a large, HEAT/Armadillo repeat–containing protein that is best known as an essential activator of the nuclear DBF2-related (NDR) kinase, Tricornered (Trc). Because *trc* is also located on chromosome 3L, we performed complementation tests between representative RE alleles and a known *trc* allele, *trc^1^*. These experiments showed that the single allele in the RE-D complementation group, *K23*, failed to complement *trc^1^*. We confirmed that the loss of Trc causes the round egg phenotype by identifying a premature stop codon in the *trc* coding region in the *K23* mutant background (Table 3) and by showing that *trc^1^* follicle cell clones can produce round eggs (data not shown). Together these data show that our screen identified two members of an NDR kinase signaling pathway regulating *Drosophila* egg shape.

As an NDR family member, Trc requires an activating phosphorylation from a Ste20-like kinase to promote its own kinase activity. Interestingly, the gene for the well-studied Ste20-like kinase Misshapen (Msn) is also located on chromosome 3L. We found that the single allele in the RE-E complementation group, *P23-5*, failed to complement two known *msn* alleles, *msn^102^ and msn^172^*. Once again, we confirmed that loss of Msn leads to a round egg phenotype by identifying a premature stop codon in the *msn* coding region in the *P23-5* mutant background (Table 3) and by showing that *msn^102^* and *msn^172^* follicle cell clones can produce round eggs (data not shown). The discovery that loss of Msn can lead to a round egg phenotype suggests that this protein might function as the upstream activating kinase for Trc in the follicle cells.

**Figure 3:**
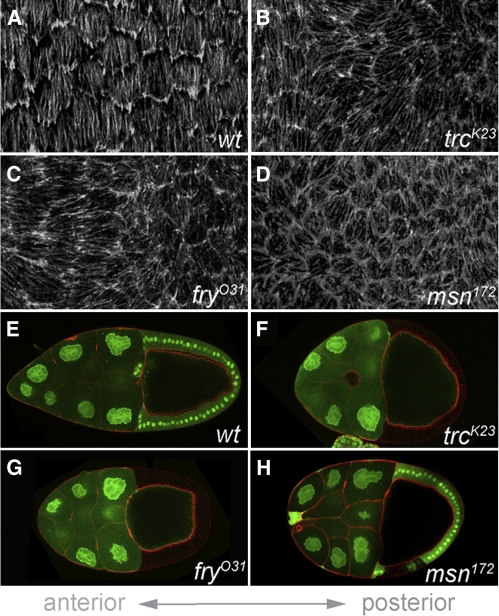
Trc, Fry, and Msn are regulators of egg chamber elongation. (A–D) Basal view of follicle cell epithelia in stage 7 egg chambers stained with phalloidin to show the planar organization of basal actin filaments. In wild-type follicle cells (A), basal actin filaments align perpendicular to the A-P axis. In contrast, when the follicle cell epithelium is predominantly composed of *trc^K23^* (B), *fry^O31^* (C), or *msn^172^* (D) mutant cells, the planar organization of basal actin filaments is disrupted. (E–H) Stage 10 egg chambers in which wild-type cells are marked with GFP (green), and all cells are outlined with phalloidin (red). A wild-type, elongated egg chamber (E) is compared with the rounded shape of egg chambers containing *trc^K23^* (F), *fry^O31^* (G), or *msn^172^* (H) mutant follicle cell clones. In all panels, anterior is to the left.

Having identified Trc, Fry, and Msn as three new egg shape regulators, we next investigated whether these proteins contribute to follicle cell planar polarity and egg chamber elongation at earlier oogenic stages. During oogenic stages 6–8, actin bundles at the basal follicle cell surface all align perpendicular to the egg chamber’s A-P axis in wild-type epithelia (Figure 3A). However, this planar organization is often lost when the epithelium is composed predominantly of *trc*, *fry*, or *msn* mutant cells (Figure 3, B–D). We have also observed that egg chambers containing *trc*, *fry*, or *msn* mutant follicle cell clones are often shorter and rounder than wild-type egg chambers (Figure 3, E–H). In stage 10 egg chambers where the epithelium is composed entirely of *trc* or *fry* mutant cells, the defect in egg chamber shape is fully penetrant but shows highly variable expressivity (data not shown). These data suggest that all three genes are required during early stages of egg chamber elongation.

**Figure 4:**
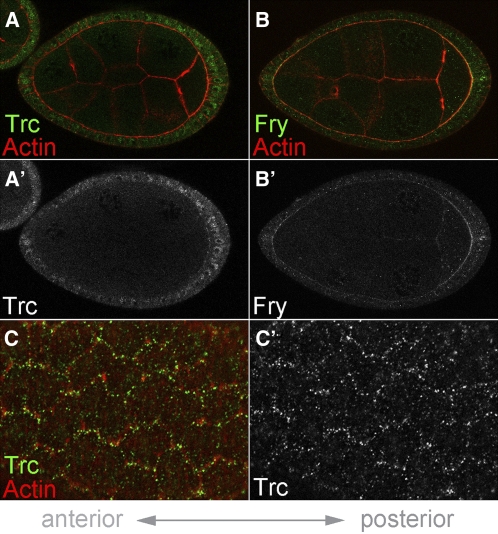
Trc and Fry protein localization in the follicle cells. (A–C) Stage 7 egg chambers, anterior to the left. (A and A′) Trc protein (green) shows diffuse staining in the follicle cell cytoplasm with some enrichment in the nuclei. (B and B′) Fry protein (green) also shows diffuse staining in the follicle cell cytoplasm with enrichment at the apical surface. (C and C′) Basal view of the follicle cells in the same egg chamber as shown in (A). Trc shows a punctate localization pattern that appears to be localized to one side of each cell. In panels A, B, and C, phalloidin marks actin in red.

To further investigate Trc and Fry’s function during the early stages of egg chamber elongation, we used previously validated antibodies to determine these proteins’ subcellular localizations in the follicle cells (Emoto *et al.* 2004; He *et al.* 2005b). When the follicle cells are viewed along their apical-basal axes during oogenic stages 6–8, Trc and Fry both show a diffuse cytoplasmic distribution, with Trc enriched in nuclei and Fry enriched at the apical surface (Figure 4, A and B). Interestingly, when the follicle cells are viewed basally, Trc shows a planar polarized distribution across the tissue, in which punctae of Trc protein appear to be localized to just one side of each cell (Figure 4, C and D). In contrast, Fry showed no obvious pattern at the basal follicle cell surface (data not shown). These observations suggest that, even though the *trc* and *fry* follicle cell phenotypes are quite similar, the localization of the two proteins do not significantly overlap.

## DISCUSSION

### Directed mosaics as a method to identify new regulators of egg chamber elongation

Since its introduction in 1998, the directed mosaic technique has been used to identify novel genes required for multiple aspects of follicle cell biology (Bilder *et al.* 2000; Denef *et al.* 2008; Duffy *et al.* 1998; Horne-Badovinac and Bilder 2008; Liu and Montell 1999; Lu and Bilder 2005; Sun *et al.* 2011; Yan *et al.* 2009, 2011). The first application of this method by Duffy *et al.* (1998) uncovered one of the first round egg mutants, likely corresponding to the βPS integrin gene *myospheroid*. However, the round egg mutants that have been studied since that time have largely been identified through reverse genetic or candidate gene approaches (Bateman *et al.* 2001; Becam *et al.* 2005; Frydman and Spradling 2001; Haigo and Bilder 2011; Viktorinova *et al.* 2009). Despite Duffy’s early discovery, it is not obvious that genetic mosaics would provide an effective forward screening method for this phenotype. Some round egg mutations like *Lar* are only partially penetrant (Bateman *et al.* 2001; Frydman and Spradling 2001). Moreover, even fully penetrant mutations like *fat2*, when studied under mosaic conditions, require large clones to produce round eggs (Viktorinova *et al.* 2009). During the execution of a broadly based follicle cell screen, we found that even a low percentage of round eggs can be detected within mosaic ovaries at the time of dissection. This simple visual assay led to the identification of 12 new round egg complementation groups. Among the four genes described in this study, *trc*, *fry*, and *msn* are essential for viability, further validating the utility of mosaic screens for identifying the function of otherwise lethal genes during oogenesis.

The elongated shape of the fly egg first came under investigation due to its dependence on an unusual mode of epithelial planar polarity (Gutzeit *et al.* 1991). Even more interest has been generated by the recent discoveries that follicle rotation and pulsed basal contractions are key components of this system (Haigo and Bilder 2011; He *et al.* 2010). However, other than the clear dependence on cell-ECM interactions, little is known about the molecular logic underlying egg chamber elongation. The round egg mutations introduced here, as well as those identified in future mosaic screens, will be invaluable resources in the pursuit of the molecular mechanisms controlling follicle cell planar polarity and egg chamber shape.

### A surprisingly high incidence of cryptic *fat2* mutations in publicly available stocks

One surprising aspect of this study was the technical difficulty we experienced in mapping the RE-A complementation group to the *fat2* gene. *fat2* encodes a protein of 4705 amino acids. The gene’s large size makes it an excellent mutagenic target, as evidenced by the six new mutant alleles produced by our nonsaturating screen. However, it was unexpected to encounter at least two independent, second-site *fat2* mutations in the course of routine complementation tests to existing stocks. Cryptic *lethal giant larvae* (*lgl*) mutations are a common contaminant in popular second chromosome stock collections (Roegiers *et al.* 2009). The high frequency of these mutations appears to be due to *lgl’s* location near the 2L telomere, which allows the gene to be easily lost through small, terminal chromosome deletions. In contrast, *fat2* is located in a more central chromosomal region, and the two cryptic lesions we identified were single base pair changes within the *fat2* coding region. Whether there is some property of the *fat2* locus that makes it particularly susceptible to sporadic mutagenesis or the mutation frequency is simply a function of the gene’s large size remains to be determined. In either case, our data reveal that even deficiencies generated in a uniform genetic background can harbor second-site mutations (Ryder *et al.* 2007).

### An NDR kinase signaling pathway regulating egg chamber elongation

The identification of both Trc and Fry in our round egg mutant collection indicates that an NDR kinase signaling pathway contributes to follicle cell planar polarity and egg chamber elongation. Trc and Fry have been previously studied in *Drosophila* for their roles in the formation of epidermal extensions like wing hairs and sensory bristles (Cong *et al.* 2001; Fang and Adler 2010; Fang *et al.* 2010; Geng *et al.* 2000; He and Adler 2002; He *et al.* 2005a,b), and in the regulation of dendritic branching and tiling patterns in larval DA sensory neurons (Emoto *et al.* 2004, 2006; Koike-Kumagai *et al.* 2009). *trc* and *fry* have identical mutant phenotypes in both tissues. Moreover, the two proteins physically interact *in vivo* (Fang *et al.* 2010; He *et al.* 2005b), where Fry promotes Trc’s kinase activity (Emoto *et al.* 2004). Despite this physical interaction, these proteins are rarely seen to overlap within cells by immunohistochemistry (Fang *et al.* 2010; He *et al.* 2005b). This apparent discrepancy has led to the hypothesis that Trc’s interaction with Fry is transient and highly regulated. Our observations of Trc and Fry in the follicle cells are consistent with this notion, as we saw no obvious co-localization between the proteins, despite the two mutant phenotypes being indistinguishable. A recent study found that truncating Trc anywhere between amino acids 338 and 404 causes the mutant protein to bind Fry more strongly than wild type (Fang *et al.* 2010). Interestingly, the C-terminal deletion caused by our newly identified *trc^k23^* allele falls in this range, suggesting that increased interaction between Trc and Fry may be deleterious to their function.

In addition to Trc’s activation by Fry, all NDR kinases must be phosphorylated by a Ste20-like kinase for full activity (Hergovich *et al.* 2006). Hippo (Hpo) phosphorylates Trc in sensory neurons (Emoto *et al.* 2006), but the activating kinase for Trc during epidermal extension formation is unknown. Hpo is unlikely to function upstream of Trc in the follicle cells, as *hpo* mutant clones do not disrupt egg chamber elongation (Meignin *et al.* 2007; Polesello and Tapon 2007; Yu *et al.* 2008). Our finding that the Ste20-like kinase Msn is required for this process raises the possibility that Msn phosphorylates Trc in this tissue. However, given that loss of Msn leads to stronger defects in egg chamber elongation than loss of Trc (data not shown), this cannot be Msn’s only function. Interestingly, Hpo directly phosphorylates both Trc and a second NDR kinase, Warts (Wts), in DA sensory neurons (Emoto *et al.* 2006). Consequently, the *hpo* dendrite phenotype is more severe than that of either *trc* or *wts* alone. It will be interesting to determine whether Msn does phosphorylate Trc in the follicle cells, and what the additional targets for this kinase are during egg chamber elongation.

The main challenge moving forward will be to identify the molecular and cellular mechanisms by which Trc promotes follicle cell planar polarity and egg chamber elongation. We have considered three likely possibilities. The first is that Trc regulates actin cytoskeletal dynamics. Epidermal extensions and dendrites are largely actin-based structures, and Trc genetically interacts with Rho-family GTPases in both contexts (Emoto *et al.* 2004; He *et al.* 2005b). Moreover, prior to hair formation, *trc* mutant pupal wing cells show increased actin levels near the apical surface (He *et al.* 2005b). Given that Trc shows a planar-polarized distribution with the actin filaments at the basal follicle cell surface, it is reasonable to believe that Trc might regulate the actin cytoskeleton in this tissue. However, we have thus far not detected any consistent actin defects in *trc* mutant follicle cells, with the exception of the loss of actin filament planar polarity common to most round egg mutants (data not shown). A second possibility is that Trc regulates one of the cellular functions ascribed to NDR kinases in other organisms, such as the regulation of centrosome duplication or transcription factor activity (Hergovich *et al.* 2007; Mazanka *et al.* 2008). Although these cellular mechanisms have not yet been linked to follicle cell planar polarity, our understanding of this process is far from complete. A third possibility is that Trc regulates the cell-ECM interactions that clearly play a central role in this system. Ultimately, distinguishing between these and other possibilities will require the identification of the direct targets of Trc kinase activity. Forward genetic screens for round egg mutants, like the one described here, provide a likely avenue for these discoveries.

## Supplementary Material

Supporting Information
