# Supplementary material for: A Screen for Round Egg Mutants in Drosophila Identifies Tricornered, Furry, and Misshapen as Regulators of Egg Chamber Elongation
Source: G3 (Bethesda). 2012 Mar 1;2(3):371–8. doi: 10.1534/g3.111.001677 (PMC3291507; doi:10.1534/g3.111.001677)
Supplement: Supporting Information [file supp_2_3_371__index.html]

Supporting Information 

# A Screen for Round Egg Mutants in *Drosophila* Identifies Tricornered, Furry, and Misshapen as Regulators of Egg Chamber Elongation

## Supporting Information for Horne-Badovinac *et al*, 2012

**Files in this Data Supplement:**

- Table S1 - Mutations on Chromosome 3L that disrupt epithelial polarity and morphogenesis in the follicle cells (PDF, 61 KB)
