# Supplementary material for: A Screen for Round Egg Mutants in Drosophila Identifies Tricornered, Furry, and Misshapen as Regulators of Egg Chamber Elongation
Source: G3 (Bethesda). 2012 Mar 1;2(3):371–8. doi: 10.1534/g3.111.001677 (PMC3291507; doi:10.1534/g3.111.001677)
Supplement: Supporting Information [file supp_2.3.371_TableS1.pdf]

Table S1 Mutations on Chromosome 3L that disrupt epithelial polarity and morphogenesis in the follicle cells

Compl. Group	Alleles	Allele Names	Notes
apical-basal polarity / multilayering			
FCP-A (*Dhc64c*)	5	*D12-5, N22-1, N37-4, N58-5, Q54-4*	Ref: Horne-Badovinac and Bilder (2008)
FCP-B (α-*Spec*)	4	*B63-5, M12, N38-2, O48-1*	
FCP-C (*avl*)	2	*N32, P56-1 (now called* avl^{3}*,* avl^{4}*)*	Ref: Lu and Bilder (2005)
FCP-D (*Gl*)	1	*K194*	Ref: Horne-Badovinac and Bilder (2008)
FCP-E	1	*P71-5*	
FCP-F	1	*Q50-4*	
epithelial gaps			
FCG-A	2	*H33-4, O50*	
FCG-B	2	*C18-5, H1-3*	
FCG-C (*rap1*)	1	*D25-4*	
FCG-D	1	*B52-3*	
FCG-E	1	*C23-4*	
FCG-F	1	*K21*	
FCG-G	1	*N104-4*	
FCG-H	1	*N105*	
FCG-I	1	*P36-1*	
FC G-J	1	*Q80-1*	
germ cell encapsulation			
FCE-A	1	*D3-2*	
FCE-B	1	*K9*	
FCE-C	1	*N81*	
FCE-D	1	*N87-4*	
FCE-E	1	*O97*	
FCE-F	1	*P27-3*	
FCE-G	1	*Q4-4*	
FCE-H	1	*Q62-1*	
other phenotypes			
FCO-A	2	*C91-1, P12-4*	long egg chambers (*phenotype not clone-associated*)
FCO-B	1	*C51-1*	wild-type cells multilayer in mosaic tissue
FCO-C	1	*M56*	follicle cells proliferate beyond stage 6
FCO-D	1	*N157*	small oocyte
FCO-E	1	*P6-4*	extensive follicle cell death
